# Effect of Pregabalin on the Development of *Sarcophaga argyrostoma* (Robineau-Desvoidy, 1830) (Diptera: Sarcophagidae) in Veterinary Forensics—Preliminary Study

**DOI:** 10.3390/insects17030255

**Published:** 2026-02-27

**Authors:** Katarzyna Czepiel-Mil, Piotr Listos, Robert Stryjecki, Ewa Pietrykowska-Tudruj, Martyna Czyżowska

**Affiliations:** 1 Department of Zoology and Animal Ecology, Faculty of Environmental Biology, University of Life Sciences in Lublin, Akademicka 13, 20-950 Lublin, Polandmartyna.czyz00@gmail.com (M.C.); 2 Department of Pathomorphology and Forensic Medicine, Faculty of Veterinary Medicine, University of Life Sciences in Lublin, Głęboka 30, 20-612 Lublin, Poland; 3 Department of Zoology, Maria-Curie Sklodowska University, Akademicka 19, 20-033 Lublin, Poland; ewa.pietrykowska-tudruj@mail.umcs.pl

**Keywords:** forensic entomology, forensic veterinary medicine, drugs, post-mortem period, pregabalin, *Sarcophaga argyrostoma*

## Abstract

Pregabalin, as the active ingredient of various medications, is used in humans to treat generalized anxiety disorder (GAD), epilepsy, fibromyalgia, and neuropathic pain. In animals, pregabalin is used as an anesthetic. The aim of the present study was to test the effect of pregabalin on the developmental parameters of *Sarcophaga argyrostoma* (Robineau-Desvoidy, 1830) (Diptera: Sarcophagidae), a fly used in forensic entomology. A culture of *S. argyrostoma* larvae was conducted on mouse carcasses. Two doses of the active substance were used in the experiment: 100 mg/kg (dose 1) and 300 mg/kg (dose 2). A control sample (without the drug) was used for comparison. The duration of the life cycle, the body weight of individual developmental stages (larvae, pupae, and adults), and their mortality were studied. The study showed that (1) pregabalin prolonged the life cycle of *S. argyrostoma*—dose 1 by two days and dose 2 by three days; (2) pregabalin caused an increase in body weight at each stage of development (larvae, pupae, and adults); (3) pregabalin caused high mortality among pupae. The highest mortality was observed in the treatment with 300 mg/kg. The preliminary results indicate that estimation of the time of death based on analysis of the developmental stages of *S. argyrostoma* on a carcass with a high content of pregabalin may be distorted relative to a case without the impact of this exogenous substance. Research on the effect of pregabalin on flies used in forensic entomology should be expanded to investigate how this compound affects the life cycles of these insects.

## Simple Summary

Exogenous substances affect the development of insects in various ways. Some shorten the natural life cycle of insects, while others extend it. The effect of exogenous substances on the duration of the life cycle is dependent on the species of insect. Pregabalin, as the active ingredient of various medications, is used in humans to treat generalized anxiety disorder. In animals, it is used as an anesthetic. The aim of the present study was to test the effect of pregabalin on the developmental parameters of *Sarcophaga argyrostoma* (Robineau-Desvoidy, 1830) (Diptera: Sarcophagidae), a fly used in forensic entomology. The duration of each developmental stage of the fly, the condition and viability of the larvae, and the body weight of each developmental stage (larvae, pupae, and adults) were determined. The results indicate that estimation of the time of death based on analysis of the developmental stages of *S. argyrostoma* on a carcass with a high content of pregabalin may be distorted relative to a case without the impact of this exogenous substance.

## 1. Introduction

Forensic entomology can be used not only to estimate the time of death, but also to determine the cause of death [1,2]. Toxicological analysis of insects collected from carcasses provides information about potential overdosing of substances, which can be the cause of death by homicide or suicide when injected or taken orally as medicines or illegal drugs [3]. Toxic substances in the body of the deceased affect the rate of appearance of necrophages [4]. Moreover, toxic substances can affect the duration of the life cycle of insects, prolonging or shortening it, which is important for precise estimation of the post-mortem interval (PMI) [5,6]. The duration of the life cycle of insects feeding on carcasses and their weight may undergo changes, which is significant for estimating the time of death [7].

One compound that can cause death in the case of overdose is pregabalin [8]. Post-mortem toxicological analyses have demonstrated pregabalin abuse in people using opioids. Analysis of German toxicological reports covering the period from 2012 to 2013 showed that pregabalin was detected in 2% of cases in the first year and 4% in the second [9].

In humans, pregabalin is used as an effective drug for controlling generalized anxiety disorder (GAD), epilepsy, fibromyalgia, and neuropathic pain [10,11,12]. In contrast to widely used benzodiazepines, pregabalin can be used in long-term treatment [13]. Improper use of pregabalin, however, can lead to complications and functional disorders [14,15,16,17]. Pregabalin is most commonly used in combination with opioids, which may increase the risk of fatal overdose. With the growing popularity of pregabalin worldwide, it is increasingly becoming a cause of death due to poisoning [18].

In veterinary medicine, pregabalin is used in dogs and cats to treat acute and chronic pain [19,20]. In addition, it can be used in domestic cats to treat stress and anxiety, in order to improve their emotional state and reduce anxiety during transport and visits to the vet [20,21,22]. Administered orally before a medical procedure, pregabalin has been used as an anesthetic in dogs and cats. It relaxes the muscles, and when it wears off, it allows for a smooth recovery without causing side effects [23]. Pregabalin alleviates pain in dogs undergoing surgery and is used to treat neuropathic pain [24,25]. Pregabalin is also used in pharmacological and toxicological experimental studies with rodents (mice and rats), e.g., to investigate its mechanism of action and analgesic effect [26,27]. In mice, the behavioural and neurochemical effects of pregabalin are studied [28].

Flies of the family Calliphoridae are one of the groups of insects commonly used as toxicological indicators in forensic investigations, but there is little data on Sarcophagidae [29]. Flies of the family Sarcophagidae utilize dead tissue at various stages of decomposition for their development, providing a good basis for the determination of minimum PMI [30]. Various species of the genus *Sarcophaga* can be used in forensic medicine [31]. These include *S. argyrostoma*, whose females lay first-instar larvae, instead of eggs, directly onto the food source (carrion or carcass) [32]. This gives the species an advantage in colonizing the carcass, and its life cycle is shorter than that of flies that lay eggs. *S. argyrostoma* larvae colonize both fresh and severely decomposed remains as well as rotting organic material, and they can also develop on human feces [33,34].

No studies have been conducted on the effect of pregabalin on the developmental parameters of Sarcophagidae species. The main goal of this research was to determine whether varied doses of pregabalin affect representatives of the family Sarcophagidae. The present study presents the results of an experiment on the effect of pregabalin on the life cycle of *S argyrostoma*. The duration of individual developmental stages of the fly, the body weight of each of the developmental stages (larvae, pupae and adults) and mortality were determined. The research hypothesis postulated that pregabalin would change the duration of the life cycle of *S. argyrostoma* relative to the control sample, would affect the weight of the larvae, pupae, and adults, and might cause high mortality in some stages. The results may provide valuable information for investigations using *S. argyrostoma* in forensic veterinary medicine and forensic entomology in cases in which a pregabalin overdose is a possible cause of death.

## 2. Materials and Methods

### 2.1. General Methods

The biological material used for the experiment came from research conducted earlier at the Department of Animal Physiology and Pharmacology in the Institute of Biological Sciences, Faculty of Biology and Biotechnology, Maria Curie-Skłodowska University in Lublin, Poland. In the original research, pregabalin had been administered to live mice intraperitoneally, after which the mice were euthanized for analysis [35]. All procedures related to the killing of animals were conducted in accordance with the applicable European Union Directive (2010/63/EU) and Polish legislation. The detailed procedure for killing the mice can be found in Nieoczym et al. [36,37] and Socały et al. [35].

The experiment presented here was conducted using mice that had been euthanized in the study referenced above. These included two dead mice, which had each received a different dose of pregabalin: 100 mg/kg (dose 1) and 300 mg/kg (dose 2). A control sample (a third mouse), which had not been treated with pregabalin, was used for comparison. Information on the pregabalin concentrations in the mice was obtained from the authors of the original research. The dead mice were exposed outdoors to attract necrophagous flies. The next day, egg packets and live L1 larvae were found on the carcasses.

### 2.2. Laboratory Experiments

The mouse carcasses with egg packets and live L1 larvae were transferred to the laboratory. We left 40 L1 larvae on each carcass, assuming that they could be *S. argyrostoma* larvae. Egg packets belonging to other fly species and the remaining L1 larvae were removed from the mouse carcasses. The three samples (dose 1, dose 2, and control) were placed in three separate plastic containers, covered with air-permeable material, and then sealed with a lid. Each container contained one dead mouse and 40 *S. argyrostoma* larvae feeding on it. A total of 120 *S. argyrostoma* larvae were used in the analysis. The species of fly that had laid the L1 larvae was preliminarily identified as *S. argyrostoma* at the L3 stage. This identification was confirmed at the adult stage. That part of the study was carried out in laboratory conditions at ~25 °C and relative humidity (RH) ~65%.

When second-instar larvae appeared, on day 3 of the experiment, the larvae were weighed for the first time. For weighing during the larval stages, 40 larvae were weighed each time in each experimental treatment (dose 1, dose 2, and control), as all the larvae survived to the pupal stage. In the case of weighing at the pupal stage, the number of individuals weighed was lower, due to high pupal mortality. Enough mouse tissue remained for the larvae to feed on until the end of the larval stage. Individual larval stages were identified based on the time elapsed and the size of the larvae [38].

During the three-week life cycle, the successively appearing developmental stages, i.e., the larvae, pupae, and adult forms of the fly, were weighed eight times at three-day intervals, using an OHAUS PIONEER analytical balance (OHAUS Corporation, Parsippany, NJ, USA). All developmental stages were weighed to the nearest hundredth of a milligram. We chose three-day weighing intervals so that the changes in the weight of the larvae would be clearly perceptible. The larvae were removed from the mouse carcasses, weighed individually, and then placed back on the carcasses. The viability and condition of the larvae were monitored daily during the experiment. At the pupal stage, the flies were transferred to separate containers and monitored until adult individuals appeared.

### 2.3. Statistical Analysis

Descriptive statistics (medians, means, sums, range, and standard deviation) were calculated using PAST ver. 4.16 [39]. The normality of the data distribution was checked by the Shapiro–Wilk test. For normally distributed data (adult body weight), one-way ANOVA was used for multiple comparisons, and Tukey’s test for different N was used as a post hoc test. For data without normal distribution (larval body weight and pupal body weight), the nonparametric Kruskal–Wallis test for multiple comparisons (H) was employed, and the non-parametric Mann–Whitney U test (Z) was used as a post hoc test. The Shapiro–Wilk, ANOVA, Kruskal–Wallis and Mann–Whitney U tests were performed, and the graphs were constructed using Statistica 13.1. The level of significance was *p* = 0.05.

## 3. Results

### 3.1. Larval Body Weight

**Figure 1:**
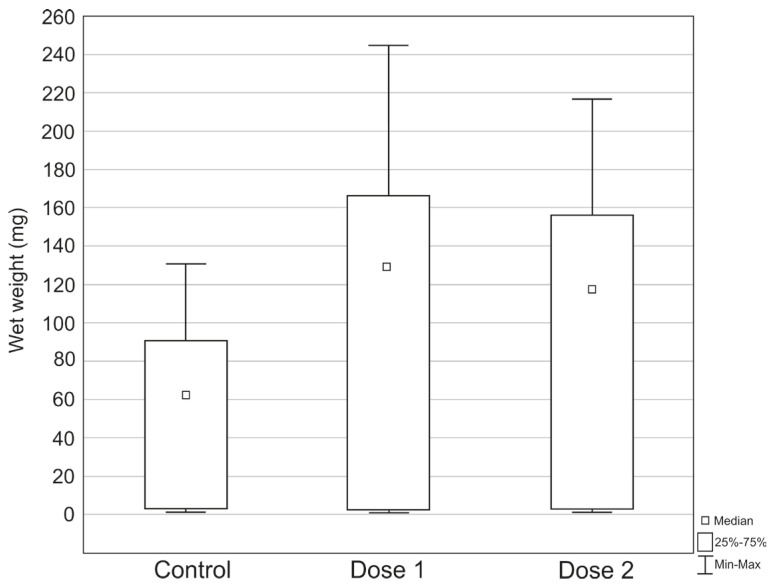
Differences in the body weight of *S. argyrostoma* larvae during the experiment.

Differences in the weight of larvae between treatments were statistically significant (H (2, N = 339) = 25.57738, *p* < 0.0001). The post hoc tests showed statistically significant differences between the control sample and dose 1 (Z = 4.79, *p* < 0.0001) and between the control sample and dose 2 (Z = 3.98, *p* = 0.0002). The highest values in larval body weight during the experiment were recorded in the treatment with dose 1 (Figure 1), i.e., the highest larval weight (244.90 mg), the highest average larval weight (105.07 mg ± 77.85), and the highest median (130.52 mg). The lowest larval body weight during the experiment was recorded in the treatment with dose 1 (0.78 mg), while the lowest average larval weight (52.05 mg ± 44.23) and the lowest median (62.58 mg) were recorded in the control sample (Figure 1).

### 3.2. Pupal Body Weight

**Figure 2:**
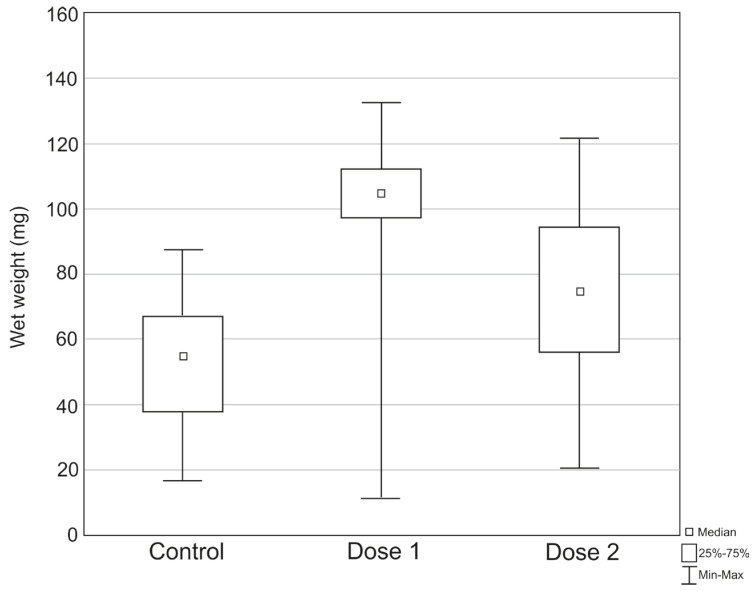
Differences in the body weight of *S. argyrostoma* pupae during the experiment.

Differences in the weight of pupae between experimental treatments were statistically significant (H (2, N = 357) = 205.2174, *p* < 0.0001). The post hoc tests showed statistically significant differences between all treatments: between the control sample and dose 1 (Z = 14.28, *p* < 0.0001), between the control sample and dose 2 (Z = 6.20, *p* < 0.0001), and between dose 1 and dose 2 (Z = 7.99, *p* < 0.0001). The highest values for pupal body weight during the experiment were recorded in the treatment with dose 1 (Figure 2): the highest pupal weight (132.56 mg), the highest average pupal weight (103.08 mg ± 14.61), and the highest median (104.80 mg). The lowest pupal body weight during the experiment (16.66 mg) was recorded in the treatment with dose 1, while the lowest average pupal weight (52.07 mg ± 17.90) and the lowest median (54.98 mg) were recorded in the control sample (Figure 2).

### 3.3. Adult Body Weight

**Figure 3:**
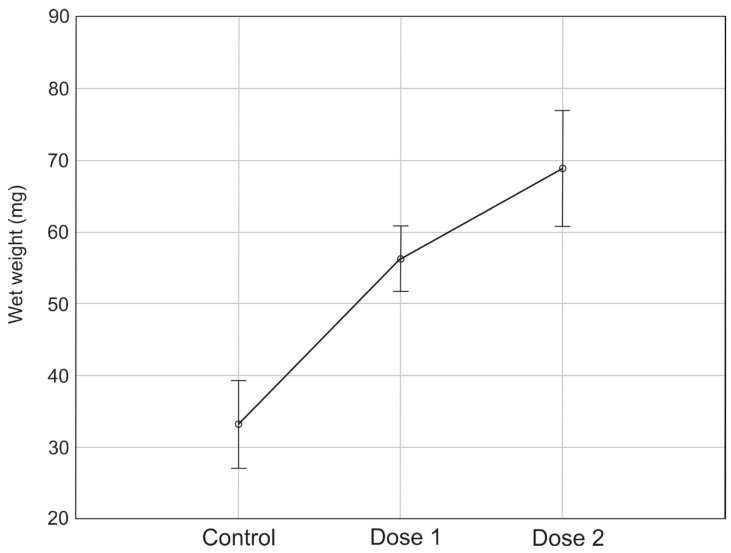
Differences in the body weight of adult flies *S. argyrostoma* during the experiment.

Differences in the weight of adult flies between experimental treatments were statistically significant (F(2, 68) = 29,184, *p* < 0.0001). The post hoc tests showed statistically significant differences between the control sample and dose 1 and between the control sample and dose 2 (*p* < 0.05). The highest values for adult body weight during the experiment were recorded in the treatment with dose 2: the highest adult weight (96.67 mg), the highest average adult weight (68.85 mg ± 14.61), and the highest median (67.35 mg) (Figure 3). The lowest values for adult body weight during the experiment were recorded in the control sample: the lowest adult weight (13.13 mg), the lowest average adult weight (33.18 mg ± 12.56), and the lowest median (36.79 mg) (Figure 3).

### 3.4. Growth Rate of S. argyrostoma

The study showed that pregabalin affected the growth rate of *S. argyrostoma* at both doses. In the treatment without the drug, the life cycle of the fly lasted from 17 to 19 days. The dose of 100 mg/kg extended the minimum duration of *S. argyrostoma* development by one day (to 18 days), and the maximum duration by two days (to 21 days). The dose of 300 mg/kg also extended the minimum duration by one day, but the maximum duration was three days longer than in the control sample (Table 1).

The longer life cycle of *S. argyrostoma* in the treatments with the drug was due to the longer development at the third-instar larval and pupal stages. The shortest development period for the third-instar larvae was the same for the control and treatment 1, amounting to four days. For the higher dose of 300 mg/kg, the minimum duration of the third-instar larval stage was two days longer than in the control and the treatment with 100 mg/kg (Table 1). The maximum duration of development of this larval stage was six days for the control, one day longer for the treatment with 100 mg/kg, and two days longer for the treatment with 300 mg/kg.

Both doses of the drug reduced the minimum duration of the pupal stage by one day in comparison with the control sample in some individuals. At the same time, in other specimens, pregabalin prolonged the pupal stage by two days (at 300 mg/kg) or three days (100 mg/kg) (Table 1).

**Figure 4:**
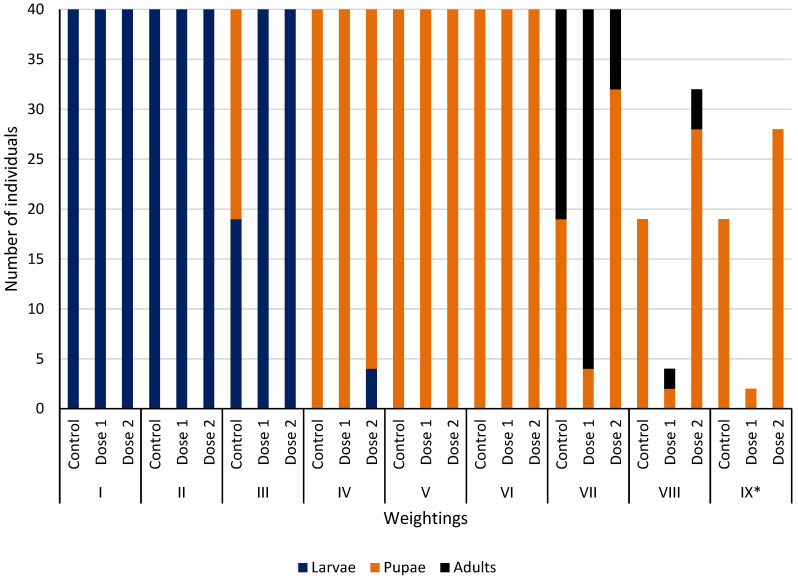
Numbers of each developmental stage of *S. argyrostoma* in each treatment during the experiment. * numbers for inspection IX indicate dead pupae.

Larvae were present in all treatments on the first, second, and third inspection days (Figure 4). On the third inspection day (day 8 of the life cycle), pupae appeared in the control sample. On the fourth day (day 11 of the life cycle), larvae were present only in the sample with the higher dose of the drug. During the fifth and sixth inspections (days 14 and 17 of the life cycle), only pupae were observed. During the seventh inspection (day 20 of the life cycle), pupae and adult forms were observed in the samples, with the most adults found in the samples with dose 1 of the drug. During the eighth inspection (day 23 of the life cycle), there were no longer any adult flies in the control sample, but they were still present in the samples with the drug. Only dead pupae were observed during the ninth inspection (Figure 4).

### 3.5. Mortality of S. argyrostoma at Each Stage of Development

All 40 individuals from each experimental treatment survived the larval period and reached the pupal stage (Figure 4). In the further stages of the experiment, high mortality was observed in the pupal stage. Out of 120 individuals used in the experiment, 49 pupae died and 71 developed into adults. The highest pupal mortality was recorded in the samples with the highest dose of the drug (300 mg/kg). In this experimental treatment, only 12 individuals developed into adults (Figure 4). All 71 individuals that reached the adult stage were free of developmental defects, irrespective of the experimental treatment.

## 4. Discussion

Necrophagous insects, especially their larvae, are of great importance in forensics. Analysis of the insect fauna colonizing a carcass, especially one in an advanced stage of decomposition, can be helpful in determining the causes and circumstances of death [40]. The detection of chemical substances that may have caused a person’s death in the bodies of necrophagous insects is important because these substances can influence the decomposition rate of the body and thus the rate of development of insects [41,42].

Various exogenous substances affect the development of insects in various ways [43,44]. Some, like cocaine, shorten the natural life cycle of insects, while others prolong it, e.g., petrol, hydrocortisone, malathion, arsenic, carbon monoxide, or hyoscine butylbromide. The effect of exogenous substances on the duration of the life cycle is largely dependent on the species of insect. Heroin and morphine have been shown to extend the life cycle of *Sarcophaga peregrina* and *Lucilia sericata* [45,46]. In *Chrysomya albiceps*, the opioid tramadol hydrochloride extended the life cycle by two days relative to the control group [47]. In *Chrysomya albiceps* and *Chrysomya putoria*, diazepam accelerated larval development [48]. Zou et al. [49] showed that ketamine shortened the larval stage in *Lucilia sericata* but extended it in *Calliphora vomitoria*. El-Samad et al. [50] showed that morphine accelerated development in *S. argyrostoma* but prolonged it in *Lucilia sericata*, *Chrysomya albiceps,* and *Chrysomya megacephala*. A study with tramadol showed that it caused faster larval and pupal development in *S. argyrostoma* than in the control sample [51]. Acceleration of the rate of development of *S. argyrostoma* larvae was also shown in a study using clonazepam [29].

The present preliminary study using pregabalin showed that the drug extended the life cycle of *S. argyrostoma* in comparison with the control. Development was prolonged in the third larval instar and then in the pupal stage. Similar results were obtained by Czepiel-Mil et al. [52] in a study on the effect of pregabalin on another fly species—*Lucilia sericata*. In that case, pregabalin also prolonged the life cycle at the stages of the third larval instar and pupa. These similar results indicate that the compound has a similar impact on life cycle duration, irrespective of the species of fly.

Exogenous substances can also have varied effects on the weight of the developmental stages of necrophagous insects. Oliveira et al. [53] showed that the analgesic and antispasmodic drug Buscopan reduced the body weight of *Chrysomya megacephala*. On the other hand, Zou et al. [49] observed an increase in the body weight of *Calliphora vomitoria* larvae feeding on a medium with ketamine. A study by Tahoun and Abouzied [51] on the effects of tramadol on the development of *S. argyrostoma* showed varied effects depending on the developmental stage: the body weight of the larvae decreased under the influence of various doses of tramadol in comparison to control larvae, while the weight of the pupae increased. GC-MS analysis showed a higher concentration of tramadol in the tissues of larvae than in pupae, which suggests that tramadol is metabolized or eliminated during the passage of the larvae into the pupal stage. An experiment by Gosselin et al. [54] using methadone and *Lucilia sericata* flies showed that the larvae eliminated metabolites very quickly. For this reason, it is not always possible to detect a foreign substance and its metabolites at this stage of development.

Exogenous substances may affect different species of necrophagous flies in different ways [5]. Varying reactions of individual fly species to the same chemical compound may result from differences in xenobiotic metabolism or from differences in intestinal microflora between species [55]. In the present preliminary study, both doses of a drug containing pregabalin caused an increase in body weight at every stage of development (larvae, pupae, and adults) of *S. argyrostoma*. The results are in contrast with the findings of Czepiel-Mil et al. [52] regarding the effect of pregabalin on another fly species, *Lucilia sericata*. In that case, all doses applied of a drug containing pregabalin reduced the body weight of the larvae, pupae, and adults of this species. The contrasting findings of the two experiments indicate that pregabalin affects the body weight of the developmental stages of flies in different ways depending on the species.

Some exogenous substances reduce the survival rate of various species of necrophagous flies. Increased mortality, especially in the larval and pupal stages, can be induced, for example, by increasing concentrations of various elements, such as cadmium (Cd), zinc (Zn), copper (Cu), antimony (Sb), barium (Ba) or lead (Pb), in the food of larvae (in *Lucilia sericata*) or by chemical substances such as thiopental (in *Calliphora vicina* larvae) or ketamine (in *Calliphora vomitoria* larvae [49,56,57,58]. Exogenous substances entering the body of insects can induce oxidative damage [29].

The present preliminary study showed that pregabalin at the higher dose increased the mortality of *S. argyrostoma* specimens in the pupal stage. However, lower pupal mortality was observed in the treatment with dose 1 than in the control sample, which may indicate that other factors than the dose of the drug contributed to this high pupal mortality. Similar results were reported by Czepiel-Mil et al. [52] in a study on the effect of pregabalin on *Lucilia sericata*. In that case, pregabalin also increased mortality in the pupae, with the highest mortality resulting from the highest dose of the drug. The similar results of the two experiments indicate that pregabalin has a similar effect on pupal mortality irrespective of the species of fly.

## 5. Conclusions

1. The exogenous substance induced the following effects:
  - Extension of the life cycle of *S. argyrostoma*. The minimum duration of the complete life cycle was 17 days in the sample without the drug, while in the samples with the drug, it was one day longer for both doses—100 mg/kg and 300 mg/kg. The maximum duration of development increased with the concentration of the drug in comparison with the control—by two days at the dose of 100 mg/kg and by three days at 300 mg/kg.
  - An increase in body weight at every stage of development (larvae, pupae, and adults).
  - High pupal mortality in the samples with the higher dose of the drug (300 mg/kg).
2. The results of the study carried out in laboratory conditions at ~25 °C and relative humidity (RH) ~65%, using dead mice as a substrate, indicate that estimation of time of death based on analysis of the developmental stages of *S. argyrostoma* on a carcass with a high content of pregabalin may be distorted relative to a case without the impact of this exogenous substance.
3. The experiment demonstrated that simple observations of developmental parameters of *S. argyrostoma* can be used for an initial diagnosis, indicating elevated concentrations of pregabalin in a carcass. Costly and complex analyses of bioaccumulation and ecotoxicology can be carried out, if necessary, at later stages of the investigation.

## Figures and Tables

**Table 1:** Duration (in days) of *S. argyrostoma* life cycle in the experimental treatments.

Stage of Development	Experimental Treatment		
	Control	100 mg	300 mg
1st-instar larva	1	1	1
2nd-instar larva	1	1	1
3rd-instar larva	4–6 *	4–7	6–8
Pupa	11	10–14	10–13
Time of cycle	17–19	18–21	18–22

* Min–max ranges refer to the duration of a given stage and may not add up to the values given for ‘duration of cycle’, which represent the minimum and maximum of the whole life cycle.

## Data Availability

The original contributions presented in the study are included in the article, further inquiries can be directed to the corresponding author.
